# A robust optimal scheduling system based on multi-performance driving for complex manufacturing systems

**DOI:** 10.1038/s41598-023-43853-w

**Published:** 2023-10-07

**Authors:** Qingyun Yu, Yaxuan Zhang, Hui Zhao, Tingyi Yu, Li Li

**Affiliations:** School of Electronic and Information Engineering, Tongji University, Shanghai, 201804 China

**Keywords:** Mechanical engineering, Electrical and electronic engineering

## Abstract

A robust optimal scheduling method driven by multi-objects is proposed for the collaborative optimization problem between dynamic scheduling, preventive maintenance of equipment, and robustness of scheduling schemes in a complex manufacturing system. Firstly, the equipment maintenance task is mapped to the process level, and composite dispatching rules with weight parameters are designed, which flexibly consider equipment maintenance and system processing status. Secondly, the performance-driven ideology is analyzed through two models based on the IWOA-MLP algorithm. Thirdly, the feedback mechanism ideology facilitates adaptive closed-loop optimizations. Finally, a series of experiments were carried out on the simulation platform of a semiconductor manufacturing enterprise in Shanghai. The experimental results show that the proposed robust optimal scheduling system can effectively deal with mixed uncertainty, improve production performances, and maintain highly robust measures.

## Introduction

The production scheduling of complex manufacturing systems involves allocating limited resources (such as equipment, facility, personnel, etc.) according to time series to achieve one or several target values^1^. As manufacturing systems are complex, random, multi-constraint, and multi-objective, their scheduling problems involve several factors, such as tasks, resources, and the amount of time it takes to perform a task. In traditional scheduling theory, all problem parameters are assumed to be fixed, but there are many types of random disturbances in the actual production process, such as unexpected equipment failures, order changes, differences among workers, and changes in dispatching rules. The manufacturing system is often unable to implement production according to the established initial scheduling scheme due to these uncertain factors in the actual production process, or the initial scheduling scheme needs to be adjusted, which will have a significant impact on production processes and outcomes. Scheduling schemes are usually required to be capable of accommodating a certain degree of random disturbance in order to ensure that the production task is successfully completed and the production effect is achieved. By slightly adjusting the scheduling scheme to make it implementable and produce satisfactory scheduling results, the scheduling scheme has a certain degree of robustness.

Therefore, the purpose of this paper is to study the adaptive closed-loop optimization approach to the multi-objective driven robust optimal scheduling of complex manufacturing systems. Specifically, this paper discusses dynamic dispatching, equipment maintenance, and robustness of scheduling schemes for complex manufacturing systems based on CT (cycle time), ODR (on-time delivery rate), EA (equipment unavailability), and RM (robustness measure). By integrating intelligent manufacturing dynamic operation optimization theory, this paper systematically solves the adaptive closed-loop optimal scheduling problem in manufacturing systems.

The arrangement of this paper is as follows. Section "Problem description" describes the proposed scheduling system. The proposed scheduling system is introduced in “Robust optimal scheduling system based on multi-performance driven”, which includes composite dispatching rules, feature selection, states-performance prediction, and other functions. “Simulation experiments” describes the simulation platform, experiment design and arrangement, and discussion of the simulation results. “Conclusions” concludes this research and future works.

## Related work

### Scheduling with equipment maintenance

Among all uncertain factors in production, machine failure is the most common. Preventive maintenance is more practical and reasonable than maintenance following machine failure and can reduce the impact of machine failure on the entire manufacturing process to a considerable extent. Maintenance scheduling in the production process refers to determining the start and duration time of each equipment maintenance task, which often takes into consideration the equipment load factor and the urgency of the maintenance task. The maintenance of production equipment and the scheduling of manufacturing processes are interrelated and interactive, but they are often studied separately^2^. The academic community has only recently begun to focus on both topics: fault maintenance are comprehensively considered in workshop management, and equipment maintenance is considered as a decision variable or constraint as part of production scheduling. By integrating optimization, production costs can be reduced, equipment life can be extended, and product quality can be improved.

Hu et al.^3^ investigated the integration of production scheduling and preventive maintenance in a two-stage hybrid flow shop, which was able to optimize both the manufacturing cycle and the availability of machines in the first stage. A strategy of scheduling preventive maintenance has been adopted by Dai et al.^4^: when reliability is reduced to the threshold value, the integrated optimization of flexible job-shop scheduling problem (FJSP) and preventive maintenance is studied. Zandieh et al.^5^ conducted an integrated optimization study on shop scheduling and preventive maintenance in FJSP by focusing on maximum completion time and system unavailability as optimization objectives. Considering the single-machine system problem, Wang et al.^6^ designed a multi-objective evolutionary algorithm based on the support vector regression proxy index to determine the processing order of a workpiece and the input of equipment maintenance resources. The flow shop scheduling problem with random failures was studied by Zhang et al.^7^. They developed two nested optimization algorithms based on the comprehensive index of quality robustness and robustness as the optimization objective. An integrated optimization model for equipment preventive maintenance and production scheduling was established by Wang et al.^8^ using discrete flow shops as the background, which improved the equipment reliability and reduced the impact of equipment failures on workpiece processing.

The existing literature indicates that production scheduling considering equipment maintenance is primarily based on single-machine systems (no tight forward or backward relationship between workpieces) and flow shops (subject to the same failure probability distribution), which are inconsistent with the actual manufacturing system. Different types of equipment often fail at different rates in complex manufacturing systems. Moreover, there are timing constraints and resource constraints between jobs, and interruptions may directly or indirectly affect the start time of other jobs via the network. Consequently, integrated scheduling optimization of complex manufacturing systems is required under uncertain random factors.

### Robust scheduling

Although research on preventive maintenance of equipment is becoming more and more sophisticated, most studies still only consider the random event of equipment failure. Other unpredictable random events occur during the production process, preventing preventive maintenance from being able to prevent all machine failures^9,10^. Various uncertain factors have led scholars to conduct a robust scheduling study of the FJSP to promote the seamless connection between production organization and equipment maintenance^11,12^.

Robust scheduling consists of defining the robustness of scheduling, specifying the robustness index and the method for measuring it. Based on this, it is also necessary to select the appropriate robust scheduling method according to the characteristics of the problem and the available information to solve the robust scheduling problem. Ba et al.^13^ measured robustness by comparing the actual scheduling goal with the initial scheduling goal, and provided a method to measure initial scheduling robustness. For scheduling problems with interval processing times, Zheng et al.^14^ used the maximum regret value of the actual scheduling target for initial scheduling under the disturbance of uncertain factors to express robustness. A decomposition method based on graph theory was proposed by Kutanoglu et al.^15^ to achieve scheduling robustness using expected average weighted tardiness as the robustness index. A dual objective Pareto optimization method was used by Xiong et al.^16^ to simultaneously optimize the performance of the FJSP scheduling and the scheduling robustness under the influence of random machine failures.

In the above documents, robustness indicators and corresponding measurement methods have been provided in part. Even so, they generally represent the ability of scheduling to maintain the original state or performance in the face of uncertainty. Only a few documents explain the criteria and basis for the design of its measurement method as well as its application and role in robust scheduling research. To make the situation more suitable for practical engineering applications, it is usually necessary to optimize the equipment maintenance centrally and dynamic dispatching as well as the robustness of the scheduling scheme to ensure that the scheduling scheme considering equipment maintenance is adaptable to dynamic processing environments, that is, robust enough to handle dynamic processing environments.

### Data driven closed loop optimal scheduling

The manufacturing industry has put forward high demands for universality, adaptability, and robustness of scheduling schemes due to the rapid development of personalized customization. Consequently, closed-loop optimal scheduling based on data-driven approaches has gradually gained a great deal of interest in academia as well as industry. It is impossible to establish a strict optimization model for the production scheduling problem, which is NP-hard, due to the impact of random factors on the robustness of the scheduling scheme. To assist in the decision-making optimization of production scheduling, a simulation system is necessary^17^.

Feng et al.^18^ proposed a production scheduling optimization method based on an intelligent factory multi-level simulation system, combining the scheduling optimization and simulation modules. Feng et al.^19^ introduced a closed loop optimization mechanism in the simulation-based hierarchical optimization modeling framework to solve the problems of scheduling implementation delay, incomplete utilization of equipment, and waste of production resources caused by the open loop implementation of traditional scheduling optimization based on the static optimization model. To adapt the scheduling rules to the characteristics of the semiconductor production line, Yu et al.^20^ proposed a self-organized scheduling method, which means that the equipment in the production line can automatically call the most effective scheduling rules that will match the optimization goal according to the current production state at any decision-making time. According to the semiconductor production line characteristics, Qiao et al.^21^ designed a combined scheduling rule. The response surface method is used to optimize the weight parameters in the scheduling rules, to improve the regulations' applicability to other production states.

Although scholars have done a lot of research on the data-based closed-loop optimal scheduling problem, the existing methods usually address specific production processes and rarely consider scheduling optimization, control integration, model consistency, and other issues. Although some methods adopt the idea of "closed loop optimization", they do not consider the universality and robustness of the scheduling scheme, let alone the integrated optimization of equipment maintenance, scheduling scheme robustness, and manufacturing system performance.

### Multi-layer-perceptron based on improved whale algorithm

Deep learning has been counted in the most popular research fields since it was proposed. Being capable of extracting potentially valuable knowledge from complex systems secures the irreplaceable important role of deep learning in big data solutions^22^. In expression prediction problems, artificial neural network is an effective method to make data modeling and is capable of fitting and representing the canonical correlation between input and output^23^.

As a typical artificial neural network, multilayer perceptron (MLP) is widely used for remote sensing and prediction of meteorological and natural data, including the combination of MLP and CNN models for atmospheric research and geological survey^24^. Theoretically, if the layers of the neural network are "deep" enough and numeric quantities of single perceptions in the hidden layer, MLP can approach arbitrary nonlinear functions even if the data distribution is very complex^25^. However, a network too deep may not be effective. Deep neural network is prone to the phenomenon of gradient appearance, and a network whose hidden layers are too more would lead to overfitting when the amount of data is not significant. Therefore, it is necessary to use some methods to train and optimize the parameters of MLP to make it more consistent with the current problem^26^.

With the development of computer science and technology, new meta-heuristic algorithms are emerging, such as the gray wolf optimization algorithm, artificial bee colony algorithm, etc. The whale optimization algorithm is a population-based meta-heuristic algorithm, which has a unique search mechanism: (1) fast convergence speed; (2) strong global search ability; (3) simple and easy to implement; (4) high stability. It has been successfully applied to model prediction and parameter optimization^27^. Therefore, the whale optimization algorithm can be used to optimize the algorithm parameters of MLP.

### Summary

Combined with the current research status, this paper will propose a joint decision-making method for production scheduling and equipment maintenance and use the multi-objective optimization method based on IWOA-MLP to build an integrated optimization system for robust scheduling and equipment maintenance, to simultaneously optimize the CT ODR, EA, and RM of scheduling schemes. Then the scheduling parameters are adaptively adjusted based on the idea of closed-loop optimization so that the scheduling rules can adapt to the current working conditions, thus effectively improving production efficiency, maintaining the availability of production equipment, avoiding the deterioration of actual scheduling performance, and maintaining the robustness of the scheduling scheme.

## Problem description

The scheduling problem of the specification $n\times m$ can be described as follows: $n$ jobs ${J}_{i}\left(i=\mathrm{1,2},\dots ,n\right)$ to be processed are processed on $m$ machines; each job ${J}_{i}$ has ${n}_{i}({n}_{i}\ge 1)$ processes, ${O}_{i,j}$ represents the $i\mathrm{th}$ process of job ${J}_{j}$; each process ${O}_{i,j}$ can be processed by one or more machines, the set of processing machines is ${M}_{i,j}\subseteq \{{M}_{1},{M}_{2},\dots ,{M}_{m}\}$; the processes on different machines have different processing times, and the processing process of each job has been determined in advance.

### Variable definition

The variables involved in this paper are defined as Table 1:

**Table 1:** variable definition.

Notations	Description
$\xi$	Random variable
${J}_{i}$	Job ${J}_{i}$,$i=\mathrm{1,2},\dots ,n$
${M}_{j}$	Machine ${M}_{j}$, j $=\mathrm{1,2},\dots m$
${M}_{0}$	A large enough positive number
${a}_{ijk}$	The job ${J}_{i}$ must be processed on the machine ${M}_{j}$ before being processed on the machine ${M}_{k}$; If yes, ${a}_{ijk}=1$; otherwise, ${a}_{ijk}=0$
${x}_{ihj}$	The job ${J}_{h}$ must be processed after the job ${J}_{i}$ is processed on the machine ${M}_{j}$; If yes, ${x}_{ihj}=1$; otherwise, ${x}_{ihj}=0$
$\xi {C}_{ij}$	Completion time of job ${J}_{i}$ on machine ${M}_{j}$
$\xi {T}_{ij}$	Processing time of job ${J}_{i}$ on machine ${M}_{j}$
$i$	Job number, $I$ denotes the collection of jobs,$I=\{\mathrm{1,2},\dots ,n\}$
$j$	Operation number, $J$ denotes the collection of operations,$J=\{\mathrm{1,2},\dots ,s\}$
${s}_{i,j}$	Starting time of job $i$ in process $j$
${t}_{i,j}$	Standard processing time of job $i$ in process $j$
${\omega }_{i,j}$	Upper limit of waiting time of job $i$ between operation $j$ and $j+1$

### Hypothesis

According to the scheduling problems of this study, the following assumptions are made:

1. The setting time of equipment, the processing preparation time, and the transportation time of the job are not considered.
2. Equipment maintenance and processing are not allowed simultaneously.
3. *n* workpieces go through $m$ processes in sequence, and each process can have multiple machines.
4. All equipment faults can be maintained, and the processing can continue to work after repair.
5. Processing tasks interrupted due to equipment failure can be continued after equipment maintenance.
6. There is no inherent priority between machines.
7. There is a specific process route between operations of the same job.

### Constraints

The scheduling problem solved in this research has the following constraints:

1 $$\begin{array}{c}\sum {a}_{ijk}=1 \forall i,j,k\wedge j\ne k\end{array}$$

2 $$\begin{array}{c}\sum {x}_{ihj}=1 \forall i,h,j\wedge i\ne h\end{array}$$

3 $$\begin{array}{c}\xi {C}_{ik}-\xi {T}_{ik}+{M}_{0}\left(1-{a}_{ijk}\right)\ge \xi {C}_{ij} \forall i,j,k\wedge j\ne k\end{array}$$

4 $$\begin{array}{c}\xi {C}_{hk}-\xi {T}_{hk}+{M}_{0}\left(1-{x}_{ihk}\right)\ge \xi {C}_{ij} \forall i,h,j\wedge i\ne h\end{array}$$

5 $$\begin{array}{c}\xi {T}_{ikM}^{st}=\mathrm{max}\left\{\left(\xi {T}_{ijM}^{st}+{T}_{ijM}^{p}\right),\xi S{T}_{ik}\right\} \forall i,j,k,r\end{array}$$

6 $$\begin{array}{c}\xi {T}_{ijM}^{et}=\mathrm{min}\left\{\left(\xi {T}_{ijM}^{st}+{T}_{ijM}^{p}\right),\left(\xi {T}_{ijM}^{st}+{\mu }_{ij}\right)\right\} \forall i,j,r\end{array}$$

7 $$\begin{array}{*{20}c} {s_{i,j + 1} - s_{i,j} - t_{i,j} \le \omega_{i,j} } \\ \end{array}$$

Formula (1) indicates that the job cannot be disassembled during processing; Formula (2) indicates that each job can only be processed by one machine at the same time; Formula (3) represents process constraints; Formula (4) represents machine constraints; Formula (5) indicates that the process ${O}_{ik}$ can only be started after the equipment $M$ completes the process ${O}_{ij}$; Formula (6) indicates that the completion time of the equipment $M$ is constrained by the processing time of the process ${O}_{ij}$ on the corresponding machine; Constraint (7) ensures that the waiting time of the workpiece between two adjacent processes cannot exceed the upper limit.

### Optimization objectives

(1) Performances of the manufacturing system.

This paper chooses the cycle time and on-time delivery rate as the optimization objectives at the scheduling level. So, the first kind of objective function is as follows:

8 $$\begin{array}{c}{f}_{1}=\mathrm{min}\left(makespan\right)=\mathrm{min}\left({C}_{ave}\right)\end{array}$$

9 $$\begin{array}{c}{f}_{2}=\mathrm{min}\left(O{n}_{time}Delivery Rate\right)=\mathrm{min}\left({\mathrm{ODR}}_{ave}\right)\end{array}$$

where, ${C}_{ave}$ denotes the average completion time of all job; ${\mathrm{ODR}}_{ave}$ indicates the average delivery rate of all job.

(2) Equipment availability.

At the level of preventive maintenance, it is assumed that all machine failures will follow the same exponential distribution, and the failure rate is ${\lambda }_{M}$. It is assumed that the machine will not fail at zero time and will return to its initial state after preventive maintenance. We adopt the equipment availability index to measure preventive maintenance's effect and take it as one of the optimization goals^22^. Based on the above assumptions, the availability of equipment $M$ at time $t$ is shown in Eq. (10):

10 $$\begin{array}{c}{A}_{M}\left(t\right)=\frac{1}{1+{\lambda }_{M}}+\frac{{\lambda }_{M}}{1+{\lambda }_{M}}\mathrm{exp}\left[-\left(1+{\lambda }_{M}\right)t\right]\end{array}$$

If $T$ is assumed to be the completion time of the previous preventive maintenance, then Eq. (11) can be used to express the availability of equipment $M$ at time $t$:

11 $$\begin{array}{c}{A}_{M}\left(t\right)=\frac{1}{1+{\lambda }_{M}}+\frac{{\lambda }_{M}}{1+{\lambda }_{M}}\mathrm{exp}\left[-\left(1+{\lambda }_{M}\right)\left(t-T\right)\right]\end{array}$$

Make $Tpoint\left(k\right)=\{0,{t}_{1},{t}_{2},\dots ,{t}_{r}, {C}_{max}\}$,${t}_{1},{t}_{2},\dots ,{t}_{r}$ represents the start time of preventive maintenance of the equipment ${M}_{k}$;$r$ represents the number of preventive maintenance; ${t}_{r+1}={C}_{max}$ represents the completion time of the last process of the equipment $k$. Since equipment availability is an increasing function, and the equipment will return to its initial state after preventive maintenance, the availability of equipment can only be calculated at time ${t}_{1},{t}_{2},\dots ,{t}_{r+1}$. Therefore, the availability of equipment can be expressed by Eq. (12):

12 $$\begin{array}{c}{EA}_{\left(k\right)}={\sum }_{i=1}^{r+1}{A}_{M}\left({t}_{i}\right)\end{array}$$

Equation (13) can indicate the unavailability of all equipment:

13 $$\begin{array}{c}EA={\sum }_{k=1}^{m}{EA}_{\left(k\right)}\end{array}$$

The availability $EA$ of all equipment shall be standardized according to formula (14):

14 $$\begin{array}{c}EA=\frac{{\sum }_{i=1}^{n}\left({EA}_{i}-{EA}_{min}\right)}{n\left({EA}_{max}-{EA}_{min}\right)}\end{array}$$

Then the third optimization objective function is shown in formula (15):

15 $$\begin{array}{c}{f}_{3}=\mathrm{min}\left(EA\right)\end{array}$$

(3) Robustness of scheduling scheme.

Robust scheduling is a scheduling method with a robustness index as the optimization objective. The higher the robustness, the closer the actual scheduling scheme is to the pre-scheduling scheme. The measurement method of the robustness index depends on uncertain factors and preferences. For the uncertain factors of discrete scenarios, the scheduling performance robustness measure $MP(\sigma )$ of scheduling $\sigma$ can have the absolute maximum regret value measure ${MPR}_{a}(\sigma )$, the relative maximum regret value measure ${MPR}_{r}(\sigma )$, the maximum (worst case) measure $MPW(\sigma )$ of scheduling objectives, and the expected scheduling objective (equal probability scenario) measure $MPE(\sigma )$. However, the above robustness measurement methods cannot reflect the dispersion degree of scheduling objectives, very poor scheduling objectives may occur in extreme scenarios, and some items in the measurement methods are difficult to solve. Therefore, this paper will adopt the method of alternative measure, and use relaxation time to express the ability of scheduling to resist the disturbance of uncertain factors, that is, to reflect the robustness.

The relaxation time between jobs can absorb the disturbance of uncertain factors in the scheduling implementation process, reduce the impact of uncertain factors on the initial scheduling, and improve the robustness of scheduling. Experiments show that scheduling robustness has a strong correlation with the average relaxation time between jobs^28^. Therefore, for the same scheduling problem, the robustness of different scheduling can be qualitatively compared by using relaxation time, and this can be used as a measure of scheduling robustness.

This study uses a relaxation-time based measurement method to describe the scheduling robustness index. For scheduling scheme $\sigma$, the robustness of the scheduling scheme is defined according to formula (16):

16 $$\begin{array}{c}{R}_{M(\sigma )}={\sum }_{i=1}^{n}\left({NS}_{{J}_{i}}\times {TS}_{{J}_{i}}\right)\end{array}$$

where, ${NS}_{{J}_{i}}$ represents the number of jobs queuing behind job ${J}_{i}$, is also the weight of job ${J}_{i}$, and represents the impact of the position of workpiece on scheduling. ${TS}_{{J}_{i}}$ represents the relaxation time of job ${J}_{i}$, that is, the difference between the latest possible start time and the earliest possible start time of job ${J}_{i}$. The greater the value of ${R}_{M(\sigma )}$, the better the robustness of the scheduling.

Standardize ${R}_{M(\sigma )}$ according to formula (17):

17 $$\begin{array}{c}RM=\frac{\left[{\sum }_{i=1}^{n}\left({NS}_{{J}_{i}}\times {TS}_{{J}_{i}}\right)\right]/n}{\mathrm{max}\left({NS}_{{J}_{i}}\times {TS}_{{J}_{i}}\right)}\end{array}$$

Then the fourth optimization objective of this research is shown in Eq. (18):

18 $$\begin{array}{c}{f}_{4}=\mathrm{max}\left(RM\right)\end{array}$$

Therefore, the multi-objective driven robust optimal scheduling scheme proposed in this paper is to take formula (8), formula (9), formula (15) and formula (18) as optimization objectives at the same time, and to optimize the robustness of the scheduling scheme on the basis of the integrated optimization of preventive maintenance and job shop scheduling, which can better balance the effectiveness and availability of resources and make the production process smoother.

## Robust optimal scheduling system based on multi-performance driven

**Figure 1:**
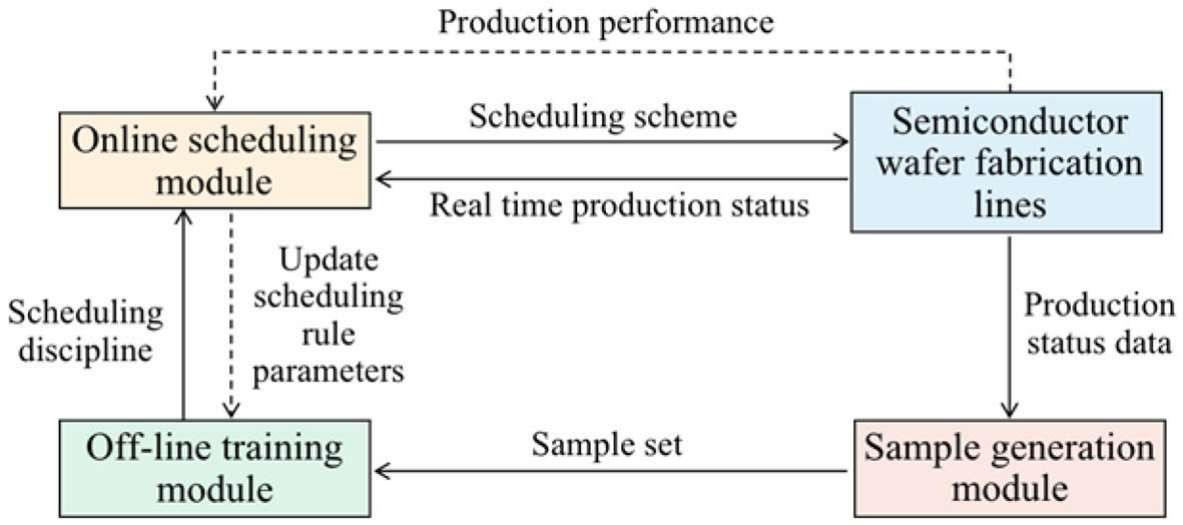
Framework of the closed-loop adaptive optimal scheduling method.

In complex manufacturing systems, if the current scheduling scheme is no longer than the optimal scheme due to disturbance factors, it is usually necessary to adopt a dynamic scheduling method to generate a new scheduling scheme to obtain better performance indicators of the production line. To solve the above problems, this paper proposes a multi-performance driven closed-loop adaptive optimal scheduling framework as shown in Fig. 1, which consists of four modules: semiconductor wafer fabrication line simulation system, sample generation module, off-line training module and online scheduling module. The basic idea is to output scheduling parameter combinations reversely based on the data related to equipment maintenance and all the production status data of the manufacturing system, driven by CT, ODR, EA, and RM. The generated scheduling scheme has a certain robustness and a certain ability to absorb and cope with uncertain disturbance and can optimize the performance index of the manufacturing system.

### Design of closed-loop scheduling system

**Figure 2:**
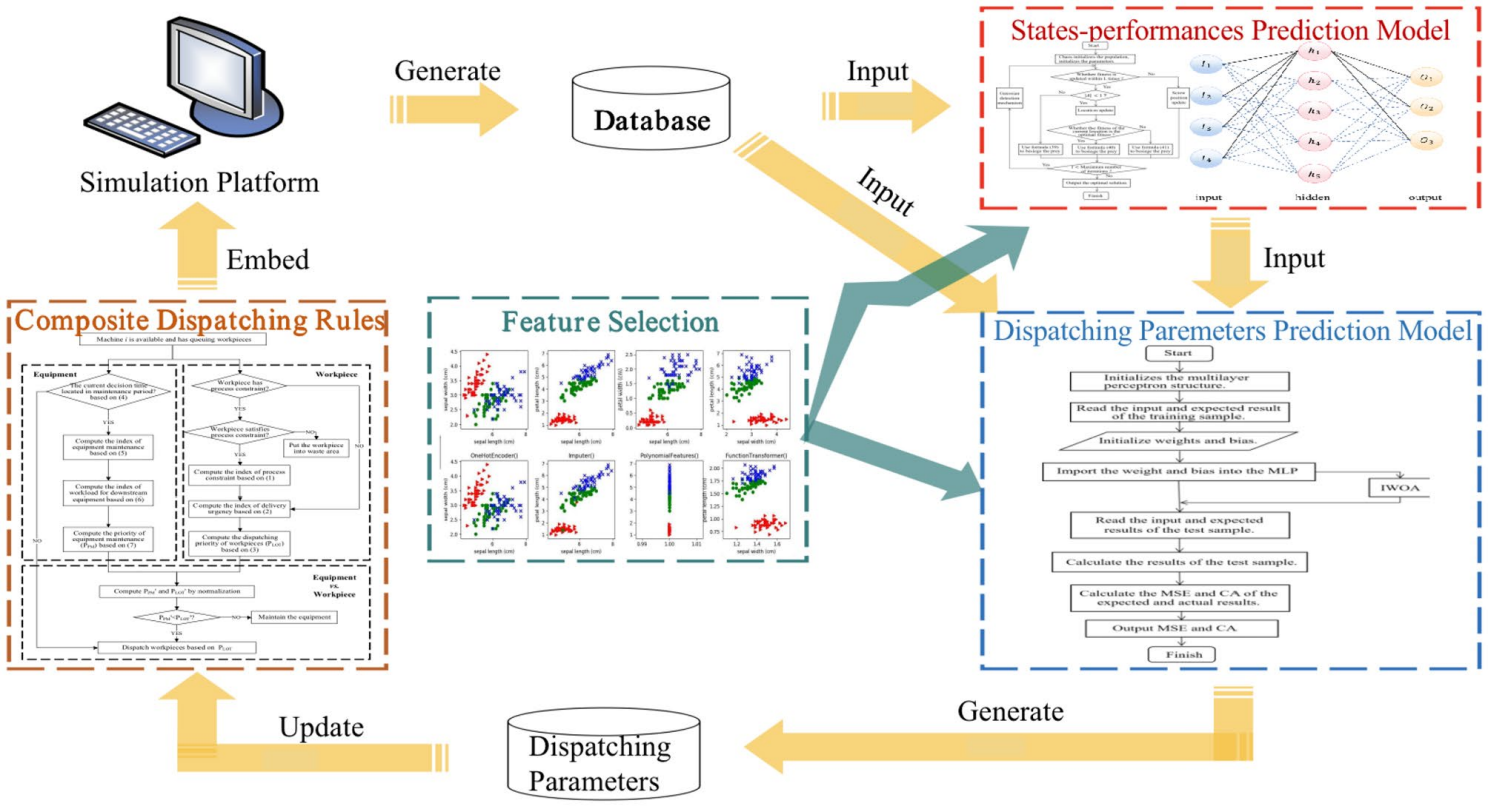
Closed-loop adaptive optimal scheduling system driven by multiple objectives.

As shown in Fig. 2, this research introduced a closed-loop feedback link into the dynamic scheduling system. The designed closed-loop optimal scheduling system driven by multi-performance mainly includes the following four parts: (1) the combined scheduling rules integrating equipment maintenance and job dispatching, (2) feature selection based on an improved immune algorithm, (3) the analysis of current production status and expected value of performance indicators through IWOA-MLP algorithm, and output the expected performance matching the current production status; (4) through IWOA-MLP algorithm, the hidden knowledge between "CT + ODR + EA + RM" and scheduling parameter combination is deeply mined, the output of scheduling parameter combination is optimized, and then the scheduling rules are updated to realize the multi-objective driven adaptive optimization process. The "simulation model" is a simulation platform built based on the actual data of a semiconductor enterprise in Shanghai.

The dispatching rule embedded in the simulation model is a combined dispatching rule, which integrates many factors, such as equipment maintenance, process constraints, workpiece priority, and urgency of workpiece delivery. Based on the simulation model, we can obtain many sample data and store them in the database. Before establishing the performance prediction model and scheduling parameter optimization model, the feature selection algorithm based on an improved immune algorithm is used to reduce the attributes of the manufacturing system, decreasing redundant features and computing time. The performance prediction model and the scheduling parameter optimization model are implemented based on IWOA-MLP. The former outputs the expected performance according to the operation state of the manufacturing system; the latter can output a combination of scheduling parameters based on the processing status and expected performance data corresponding to excellent samples. The newly generated scheduling parameter combination is updated to the combined scheduling rules to achieve optimal adaptive scheduling.

### Composite dispatch rules

**Figure 3:**
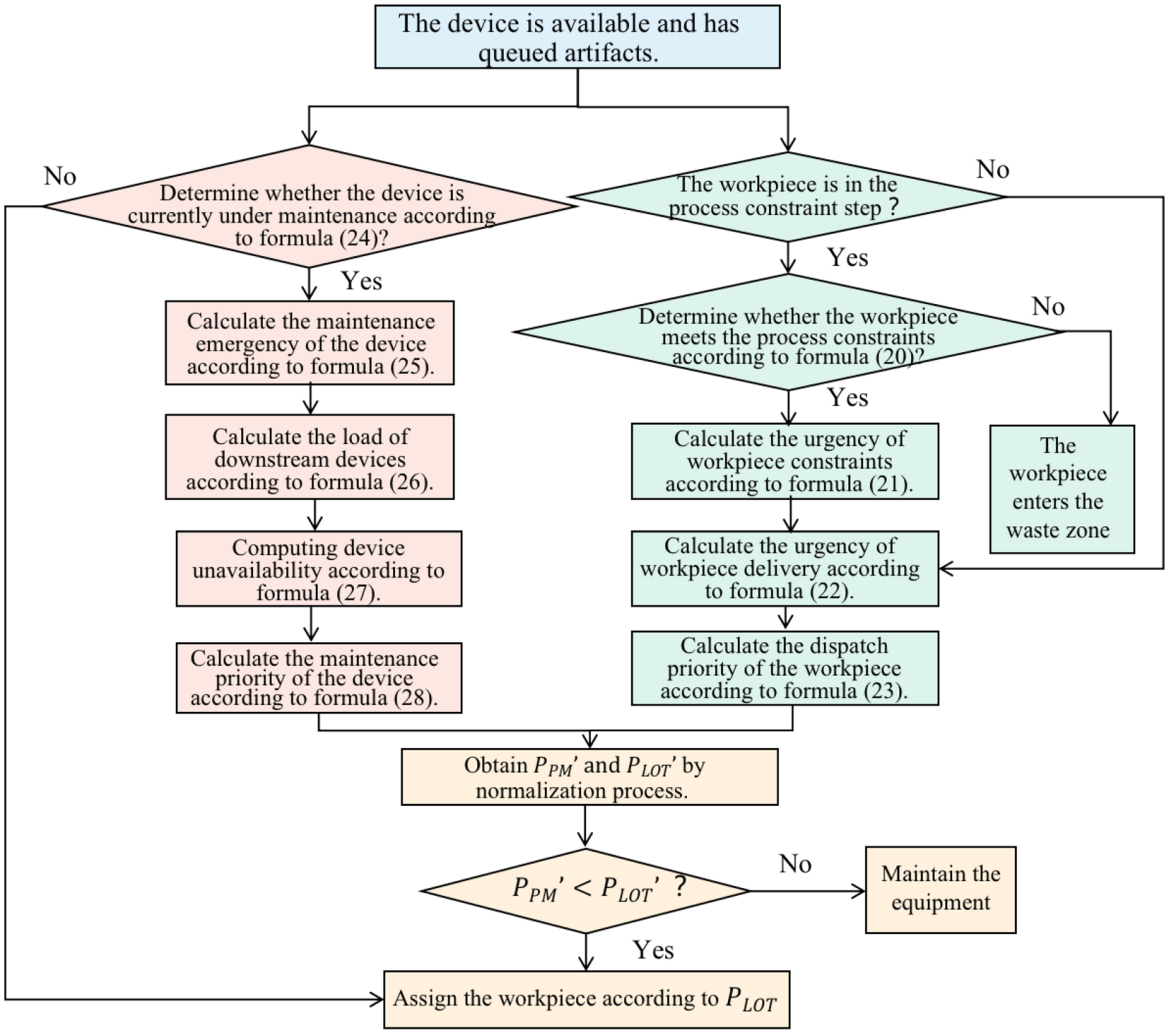
Combined scheduling rules considering equipment and workpiece.

The traditional heuristic dispatching rule does not consider process constraints, which makes the satisfaction rate of process constraints of the production line low. Then leads to the reduction of the ODR of the workpiece. This paper designs a semiconductor production line scheduling rule that comprehensively considers flexible maintenance of equipment, process constraints, and dynamic dispatching. It will consider multiple factors related to process constraints and factors related to flexible maintenance of equipment and job dispatching, such as downstream equipment load, equipment maintenance urgency, job delivery urgency, and equipment availability. The scheduling rule process is shown in Fig. 3:

Step 1: Judge whether the workpiece meets the process constraint according to formula (19). If yes, go to step 2; otherwise, put the workpiece into the scrap area.

19 $$\begin{array}{c}\left\{\begin{array}{c}{G}_{n,J}^{j}<{H}_{J}-{T}_{n}^{J}, meets the process constraint \\ {G}_{n,J}^{j}\ge {H}_{J}-{T}_{n}^{J}, violates the process constraint\end{array}\right.\end{array}$$

Step 2: Calculate the process constraint urgency ${P}_{1}$ of the workpiece according to Eq. (20).

20 $$\begin{array}{c}{P}_{1}=\left\{\begin{array}{c} MAX {G}_{n,J}^{j}\times O<{H}_{J}-{T}_{n}^{J}\\ \frac{{G}_{n,J}^{j}\times O}{{H}_{J}-{T}_{n}^{J}} {G}_{n,J}^{j}\times O<{H}_{J}-{T}_{n}^{J}\end{array}\right.\end{array}$$

Formula (20) means that at time $t$, for the workpiece with process constraints, the greater the ratio between the theoretical value and the actual value of the remaining processing time of the process constraints, the greater the probability that the process constraints will not be satisfied in the Step 3, and the easier it is to be selected by the equipment for priority processing; If the theoretical process constraint remaining processing time multiplied by the time constant $O$ is greater than the actual process constraint remaining processing time, then the probability of the workpiece not meeting the process constraint is exceptionally high. The system may mark it as an urgent workpiece, that is, it has the highest priority on the equipment.

Step 3: Calculate the delivery urgency of the workpiece according to Eq. (21).

21 $$\begin{array}{c}{P}_{3}=\frac{\sum {L}_{n}}{{D}_{n}-{T}_{NOW}}\end{array}$$

where, $\sum {L}_{n}$ represents the remaining processing time of the workpiece, ${D}_{n}$ represents the theoretical delivery date of the workpiece (one of the known attributes of the workpiece, expressed in a certain time), and ${T}_{NOW}$ represents the current decision time.

Step 4: Calculate the work piece dispatching priority according to Formula (22).

22 $$\begin{array}{c}{P}_{LOT}={\alpha }_{1}\cdot {P}_{1}+{\alpha }_{2} \cdot {P}_{2}+{\alpha }_{3} \cdot {P}_{3}\end{array}$$

where, $({\alpha }_{1},{\alpha }_{2},{\alpha }_{3})$ denote the three random parameters of the scheduling algorithm; ${P}_{2}$ is the customer priority of the piece (one of the known attributes of the workpiece, whose value is a number between 1 and 4). Considering the different orders of magnitude of ${P}_{1}$, ${P}_{2}$ and ${P}_{3}$, the value intervals of the three random numbers $({\alpha }_{1},{\alpha }_{2},{\alpha }_{3})$ are different. The value intervals of ${\alpha }_{1}$ and ${\alpha }_{3}$ are (0–1), and value intervals of ${\alpha }_{2}$ is (0–0.25).

Step 5: Determine whether the current time is in the maintenance period of the equipment according to formula (23). If the current time is in the equipment maintenance period, go to step 6; otherwise, dispatch the workpieces according to the value of ${P}_{LOT}$.

23 $$\begin{array}{l}\left\{\begin{array}{l}{T}_{NOW}-{T}_{PM-S}\ge 0 \\ {T}_{PM-E}-{T}_{NOW}\ge {T}_{PM-MAX}\end{array}\right.\end{array}$$

where, ${T}_{NOW}$ represents the current decision-making time; ${T}_{PM-S}$ represents the earliest start time of equipment maintenance; ${T}_{PM-E}$ represents the latest end time of equipment maintenance; ${T}_{PM-MAX}$ represents the maximum maintenance time of the equipment.

Step 6: Calculate the emergency degree ${P}_{4}$ of equipment maintenance according to formula (24).

24 $$\begin{array}{c}{P}_{4}=\frac{{T}_{PM-MAX}}{{T}_{PM-E}-{T}_{NOW}}\end{array}$$

Step 7: Calculate the load degree ${P}_{5}$ of downstream equipment according to Eq. (25).

25 $$\begin{array}{c}{P}_{5}=\sum \frac{{p}_{id}^{n}}{{T}_{id}}\end{array}$$

where, ${p}_{id}^{n}$ represents the occupation time of workpiece $n$ on downstream equipment $id$; ${T}_{id}$ represents the processing capacity of downstream equipment $id$ on that day. Formula (25) indicates that the heavier the equipment load is, the higher its information variable is. If ${\tau }_{i}^{n}(t)\ge 1$, it indicates that the load of the device has exceeded its available time, and the device is marked as a bottleneck device. It should be noted that if there are multiple equipment that can complete a specified operation of a workpiece, ${T}_{id}$ represents the sum of the processing capacity of this type of equipment.

Step 8: Calculate the unavailability of equipment according to Formula (26) and Formula (14).

26 $$\begin{array}{c}{P}_{6}=BM\end{array}$$

Step 9: Calculate equipment maintenance priority ${P}_{PM}$ according to formula (27).

27 $$\begin{array}{c}{P}_{PM}={\alpha }_{4}\,\cdot\, {P}_{4}+{\alpha }_{5}\,\cdot\, {P}_{5}+{\alpha }_{6}\,\cdot\, {P}_{6}\end{array}$$

where, $({\alpha }_{4},{\alpha }_{5},{\alpha }_{6})$ represents three random parameters of the scheduling algorithm.

Step 10: Normalize the dispatching priority of the workpiece ${P}_{LOT}$ and the equipment maintenance priority ${P}_{PM}$, and record the results as ${P}_{LOT}{\prime}$ and ${P}_{PM}{\prime}$ respectively.

Step 11: Compare the normalized job scheduling priority ${P}_{LOT}{\prime}$ with the equipment maintenance priority ${P}_{PM}{\prime}$'; If ${P}_{LOT}{\prime}>{P}_{PM}{\prime}$, perform job scheduling; Otherwise, perform equipment maintenance. The duration of equipment maintenance is set to the random number duration in the interval $({T}_{PM-MIN}, {T}_{PM-MAX})$.

### Multi-layer perceptron model based on improved whale optimization algorithm training

#### Whale optimization algorithm

The whale optimization algorithm is a swarm intelligence optimization algorithm developed by simulating humpback whales' bubble net foraging mode. The algorithm mainly consists of two stages: shrink and surround and position update.

(1) Shrink surround.

Whales can sense and surround their prey. Because the position of the optimal design in the hunting or search space is inconsistent with the previous position, the WOA optimization algorithm assumes that the current optimal candidate solution is the target prey or close to the optimal solution. In this case, the whale defines the best search agent; then, other search agents will try to change their positions and move closer to the best search agent. The hunting behavior of shrinking enclosure is described by the following formula:

28 $$\begin{array}{c}X\left(t+1\right)=X*\left(t\right)-A\,\cdot\, {D}_{1}\end{array}$$

29 $$\begin{array}{c}{D}_{1}=\left|C\,\cdot\, {X}^{*}\left(t\right)-X\left(t\right)\right|\end{array}$$

$t$ represents the current iteration number; $A$ and $C$ are vector coefficients; $X\left(t\right)$ represents the current time position; $X\left(t+1\right)$ represents the next time position; ${D}_{1}$ is the absolute value of the difference between $C$ times the prey position and the current whale position, and ${X}^{*}\left(t\right)$ is the position vector of the current optimal solution. If there is a better solution for each iteration result, that is, the fitness value of the position at this time is less than the fitness value of ${X}^{*}\left(t\right)$, then the whale position vector at this time is set to a new ${X}^{*}$.

The calculation formula of $A$ and $C$ is as follows:

30 $$\begin{array}{c}A=2\,\cdot\, a\,\cdot\, {r}_{1}-a\end{array}$$

31 $$\begin{array}{c}C=2\,\cdot\, {r}_{2}\end{array}$$

32 $$\begin{array}{c}a=2-\frac{2t}{{T}_{max}}\end{array}$$

${r}_{1}$ and ${r}_{2}$ are random numbers within [0,1], and ${T}_{max}$ is the maximum number of iterations. In this paper, the value of ${T}_{max}=500$, and the value range of $a$ is [0,2]. It changes linearly with the increase of $t$.

(2) Location update.

There are two ways to explore and update whale position, one is spiral position update, the other is random search. To simulate the position update mode of whales at a certain time, ensure that whales have equal probability to choose spiral position update or random search mode at the same time. Set the random number $p$ whose value range is [0, 1]. The update method of whale position is selected randomly by the size of p value.

When $p\ge 0.5$, choose the method of spiral position, and establish the spiral position equation to update the next whale position by simulating the way the whale spiral surrounds its prey. The calculation formula is as follows:

33 $$\begin{array}{*{20}l} {X\left( {t + 1} \right) = D_{2} \cdot e^{{bl}} \cdot \cos \left( {2\pi l} \right) + X^{*} \left( t \right)} \\ \end{array}$$

34 $$\begin{array}{c}{D}_{2}=\left|{X}^{*}\left(t\right)-X\left(t\right)\right|\end{array}$$

35 $$\begin{array}{c}{a}_{1}=-1-\frac{t}{{T}_{max}}\end{array}$$

36 $$\begin{array}{c}l=\left({a}_{1}-1\right)*{r}_{3}+1\end{array}$$

where, ${D}_{2}$ represents the distance between the prey and the whale; $b$ represents the parameter controlling the spiral shape, which is set as 1 in this paper; ${a}_{1}$ is a linear change parameter within [-2, -1]; ${r}_{3}$ is a random number between [0, 1]; the value range of $l$ is [-2, 1].

When $p<0.5$, choose the random search position method. The random search is divided into two ways. When $\left|A\right|<1$, it means that the whale is moving toward the prey position. Currently, contraction and enclosure formula is used to simulate the action of the whale, that is, use formula (29) to surround the prey.

When $\left|A\right|\ge 1$, it means that the whale moves beyond the position where the prey exists. At this time, the whale will give up the previous moving direction and search for new updated positions in other directions randomly to avoid falling into local extreme value. As shown in Eqs. (37) and (38).

37 $$\begin{array}{c}{D}_{rand}=\left|C\,\cdot\, {X}_{rand}\left(t\right)-X\left(t\right)\right|\end{array}$$

38 $$\begin{array}{c}X\left(t+1\right)={X}_{rand}\left(t\right)-A\,\cdot\, {D}_{rand}\end{array}$$

where, ${X}_{rand}$ represents the randomly selected whale position vector, and ${D}_{rand}$ represents the absolute value of $C$ times the difference between ${X}_{rand}$ and $X\left(t\right)$.

#### Improved whale optimization algorithm

**Figure 4:**
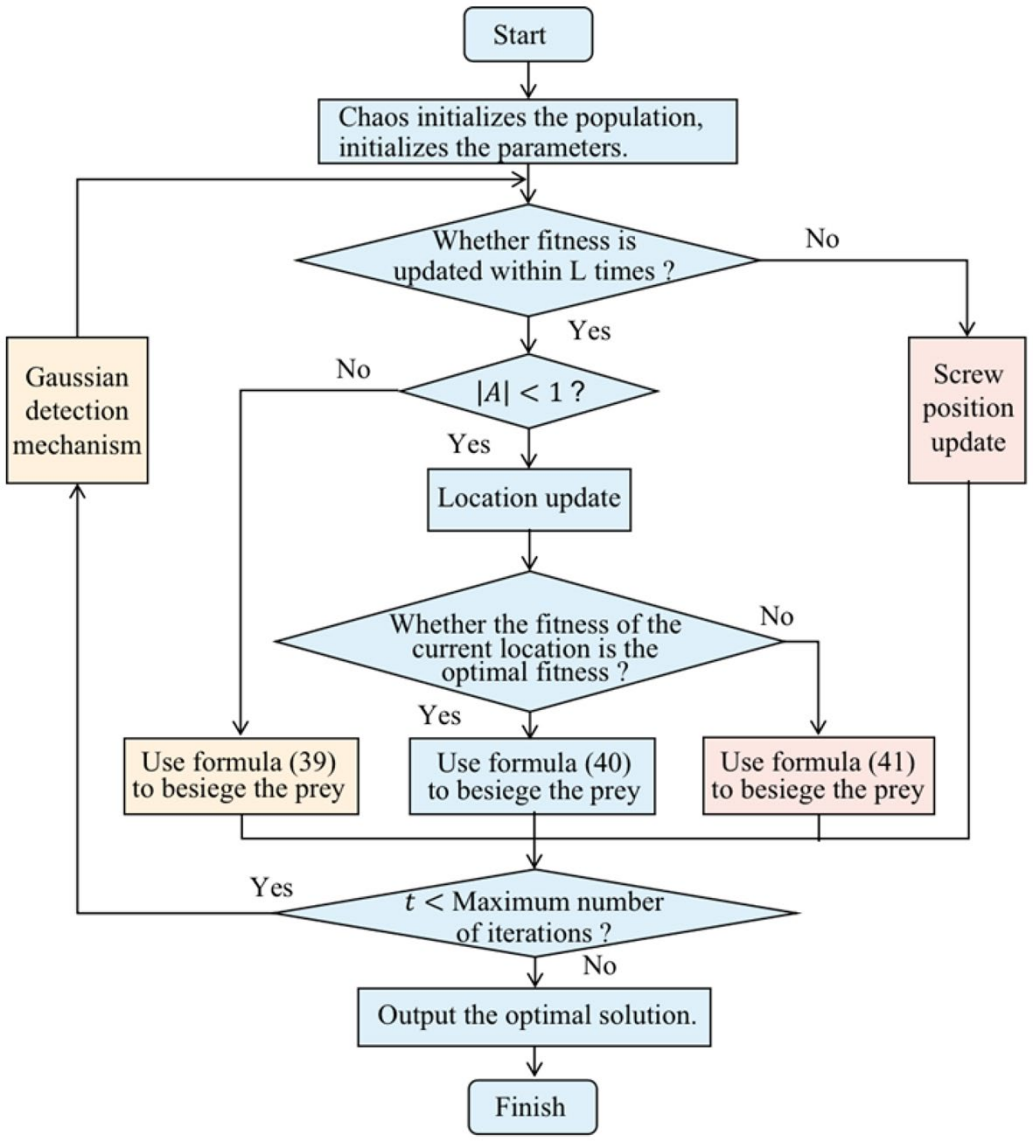
Improved whale optimization algorithm.

In the basic whale optimization algorithm, the whale position update process is through the random selection of position update mechanism, so there is a problem that the most effective update method cannot be selected in the whale position update; Moreover, in the search process of the algorithm, there is a problem that multiple iterations do not change the leader ${X}^{*}(t)$ position, leading to the premature end of the convergence process, that is, when solving the optimization problem, it may quickly converge to the local optimal, and ultimately reduce the quality of the solution of the optimization algorithm. Aiming at the problems in the traditional whale optimization algorithm, this paper proposes the improved whale optimization algorithm IWOA based on the siege mechanism, as shown in Fig. 4, and the details are as follows:

1. The initial population position of the algorithm is randomly generated by chaotic Tent mapping to make the population distribution more uniform and accelerate the convergence speed of the algorithm.
2. A new nonlinear parameter $a$ is proposed, which makes the whale optimization algorithm adapt to complex nonlinear problems.
3. The fitness control mechanism is introduced to prevent the stagnation of update and improve the ability of the algorithm to jump out of the local optimum by controlling the population position update.
4. Introducing the Harris Hawk siege mechanism to speed up the hunting of whales.
5. At the end of each whale hunting iteration, the position control mechanism of Gaussian detection is added to increase the optimization accuracy of the algorithm.
  39 $$\begin{array}{c}Y={X}^{*}\left(t\right)-A\,\cdot\, {D}_{1}\end{array}$$
  40 $$\begin{array}{c}Z=Y+S*LF\left(D\right)\end{array}$$

#### Training multi-layer perceptron based on improved whale optimization algorithm

Multilayer perceptron, known as Deep Neural Network (DNN), is the simplest neural network structure, which is composed of input layer, hidden layer, and output layer. Each layer consists of multiple neurons. Although its structure is simple, it can learn the deeper nonlinear characteristics of data. And it is suitable for such a huge nonlinear system, i.e., a complex manufacturing system.

**Figure 5:**
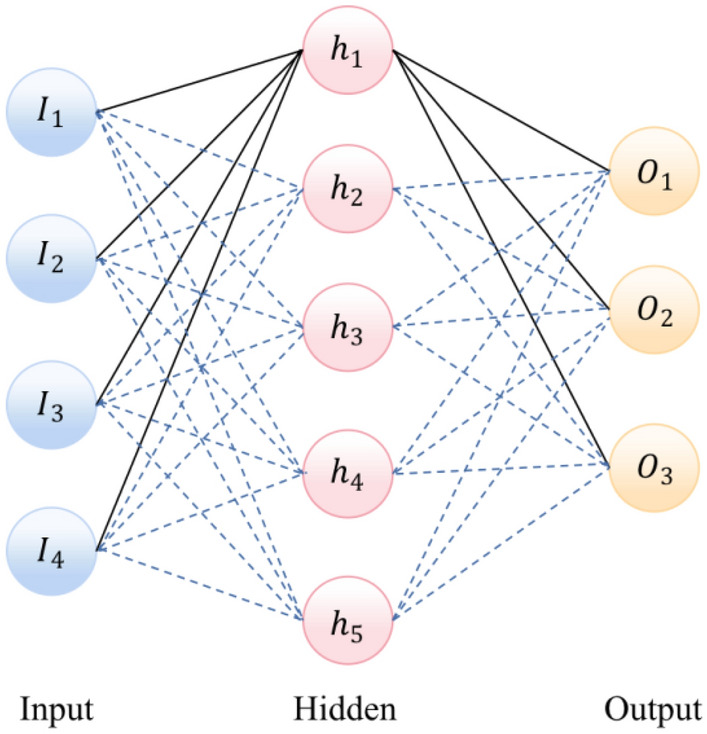
Structure diagram of multi-layer perceptron.

As shown in Fig. 5, MLP layers are fully connected. The bottom layer is the input layer, the middle layer is the hidden layer, and the last layer is the output layer. The input layer neurons are responsible for receiving information. If an n-dimensional vector is input, there are $n$ neurons. The hidden layer neurons are responsible for processing the input information. First, it is fully connected with the input layer. Assuming that the input layer is represented by a vector $X$, the calculation form of the hidden layer output is:

41 $$\begin{array}{c}f\left({W}_{1}X+{b}_{1}\right)\end{array}$$

where, ${W}_{1}$ is the connection coefficient (weight matrix), ${b}_{1}$ is the offset vector, and the function $f(Function)$ can be $Sigmoid$, that is, $sigmoid(a)=1/(1+{e}^{-a})$; or the function $tanh$, that is, $tanh(a)=({e}^{a}-{e}^{-a})/( {e}^{a}+{e}^{-a})$.

**Figure 6:**
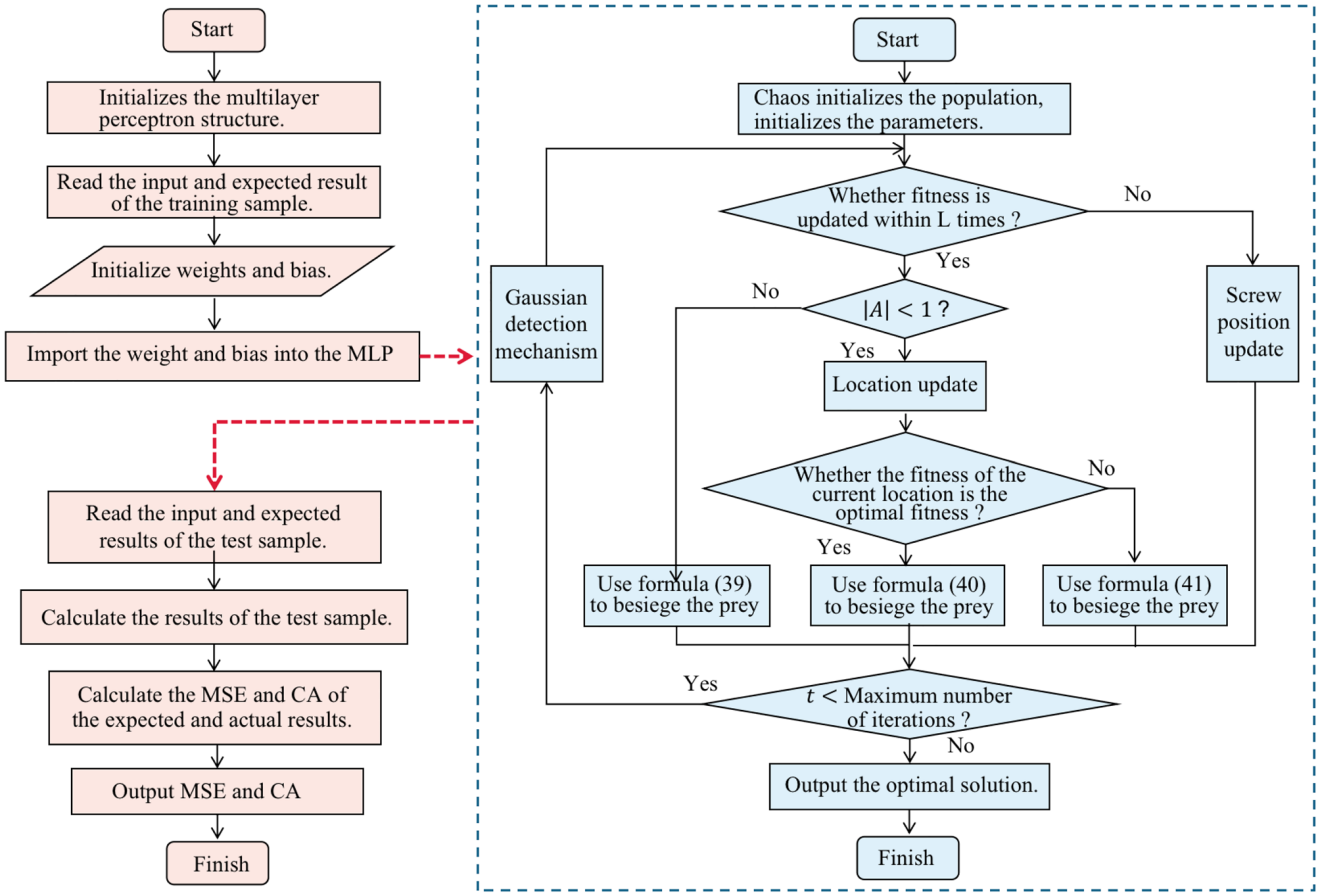
Flow chart of the IWOA-MLP algorithm.

The output layer neurons are responsible for the computer's cognition of the input information. The hidden layer to the output layer can be regarded as a multi category logical regression (i.e., $Softmax$ regression). Therefore, the output of the output layer is $Softmax({W}_{2}X+{b}_{2})$, and ${X}_{1}$ represents the output $f({W}_{1}X+{b}_{1})$ of the hidden layer. The above three-layer MLP model can be summarized as follows:

42 $$\begin{array}{c}f\left(x\right)=f\left({b}^{\left(2\right)}+{W}^{\left(2\right)}\left(S\left({b}^{\left(1\right)}+{W}^{\left(1\right)}x\right)\right)\right)\end{array}$$

where, $f$ is the function $Softmax$. Therefore, all parameters of MLP model are the connection weight matrix $W$ and offset vector $b$ between layers, including ${W}_{1}$, ${b}_{1}$, ${W}_{2}$, and ${b}_{2}$. For specific problems, the determination of these parameters is the optimization problem of solving the best parameters. This paper uses IWOA to train MLP parameters. The IWOA-MLP process is illustrated in Fig. 6.

## Simulation experiments

Since there is no standard example of a multi-objective flexible job shop robust scheduling model in the literature at home and abroad, to test the effectiveness of the scheduling model constructed and the solution strategy designed, this paper selects a simulation platform of a semiconductor manufacturing enterprise in Shanghai to verify. The company is one of the largest chip processing plants in China, mainly engaged in large-scale integrated circuit chip manufacturing. It is a typical complex manufacturing system with a large scale, complex process flow, and high reentry. The manufacturing system has 5- and 6-inch mixed production lines, four processing methods (single-chip processing, batch processing, multi-chip processing and slot processing), more than 800 equipment, 10 processing zones, hundreds of products, and thousands of processes flow. The monthly output of 5-inch chips is up to 28,000 pieces, and the monthly output of 6-inch chips is up to 51,000 pieces.

### Experiments description

Combined with the actual situation of the semiconductor production line and the needs of the enterprise, this paper selects four performance indicators as the objectives of scheduling optimization: average CT of the workpiece, ODR of the workpiece, EA, and RM of the scheduling scheme^30^. To analyze the effectiveness of the scheduling system proposed in this paper, we design simulation experiments from multiple dimensions. The experimental design mainly includes the following three parts.

(1) Comparison of compound scheduling rules with heuristic rules and other dispatch rules.

In this paper, we improve the scheduling rules for the two bottleneck processing areas, the photolithography area, and the oxidation area, and adopt the combined scheduling rules that comprehensively consider the equipment and the workpiece. The average CT, ODR, EA, and RM of the scheduling scheme is counted. To evaluate the advantages and disadvantages of combined dispatching rules more intuitively, a variety of comparison experiments are set up for comparison: FIFO, EDD, SPT and the adaptive dispatching rules mentioned in the literature^29^.

(2) Closed-loop optimization verification based on IWOA-MLP algorithm.

**Figure 7:**
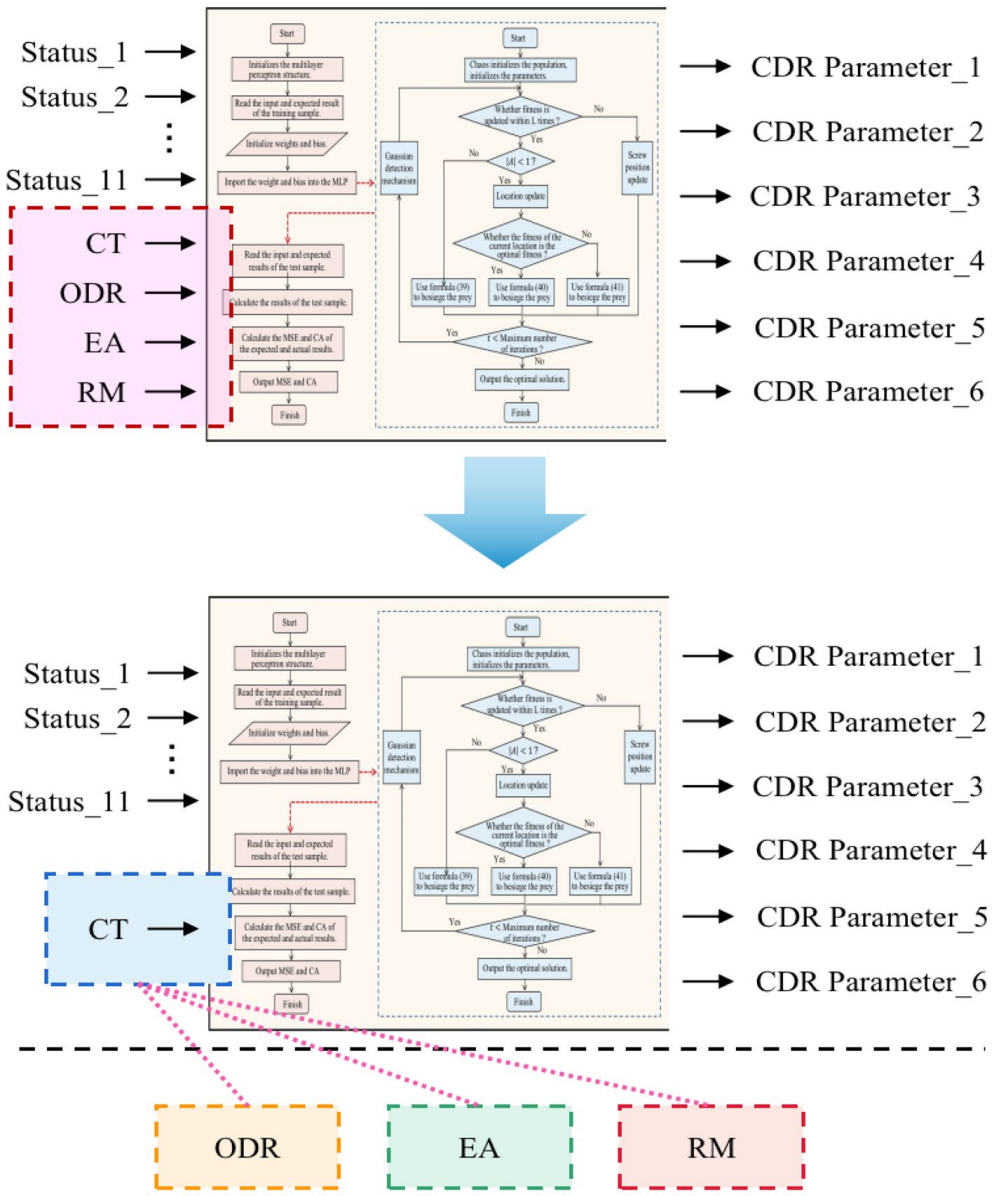
Closed-loop optimization based on different driving sources.

To test the effectiveness of the designed "closed-loop optimization based on IWOA-MLP algorithm" link, we conducted simulation experiments and comparisons between the combined scheduling rules and the entire scheduling system. As shown in Fig. 7, to analyze the "closed-loop optimization" from multiple dimensions, "driving sources" will be selected from different perspectives: CT (CT refers to the time span from when a wafer is put into the production line to when all procedures of the wafer are completed), ODR (ODR denotes the ratio of the number of the on-time delivered wafers to all completed wafers, which can reflect the completion degree of production tasks), EA (EA refers to the ratio of the actual processing time to the available time of the equipment), and RM (a performance which used relaxation time to express the ability of scheduling to resist the disturbance of uncertain factors).

(3) Comparison of this scheduling system with the self-organizing scheduling system.

Also based on the simulation platform of a semiconductor manufacturing enterprise in Shanghai, simulation experiments were conducted on the scheduling system proposed in this paper, the self-organizing scheduling system proposed in work^20^ and the predictive scheduling system proposed in work^31^ respectively. Analyze and compare the average CT of workpieces, ODR, EA, and RM of scheduling schemes.

### Analysis of experimental results

Corresponding to the experimental description part, this part will analyze the results of simulation experiments from three aspects: "combined scheduling rules", "closed-loop optimization", and "scheduling system". Before this, we first make a brief analysis of the attribute selection results of offline work. The production status attribute of the manufacturing system can describe the processing status of the workpiece and the running status of the equipment in the processing area, which can well reflect the real-time running status of the semiconductor production line. This paper selects the attribute selection method based on the improved immune algorithm to screen the attributes of the manufacturing system and selects the 11 attributes that strongly correspond to the performance indicators from the 67 attributes of the manufacturing system. The results are shown in Table 2.

**Table 2:** Selected features.

No	Selected features
1	The number of WIP in 5 "production line
2	The ratio of the WIP quantity in oxidation area to that in the whole manufacturing system
3	The ratio of the WIP quantity in photography area to that in the whole manufacturing system
4	The ratio of the WIP quantity in dry collecting area to that in the whole manufacturing system
5	The ratio of the number of bottleneck equipment to that of the whole manufacturing system
6	The ratio of the number of available bottleneck equipment in oxidation and diffusion area to that in the whole manufacturing system
7	The ratio of the number of available bottleneck equipment in photography area to that in the whole manufacturing system
8	The ratio of the number of workpieces processed by the manufacturing system to the throughput
9	Average remaining processing time of workpieces
10	Number of urgent workpieces (hot lots) in the whole manufacturing system
11	Number of urgent workpieces in oxidation and Photography Area

#### Combined scheduling rule considering device and workpiece

**Figure 8:**
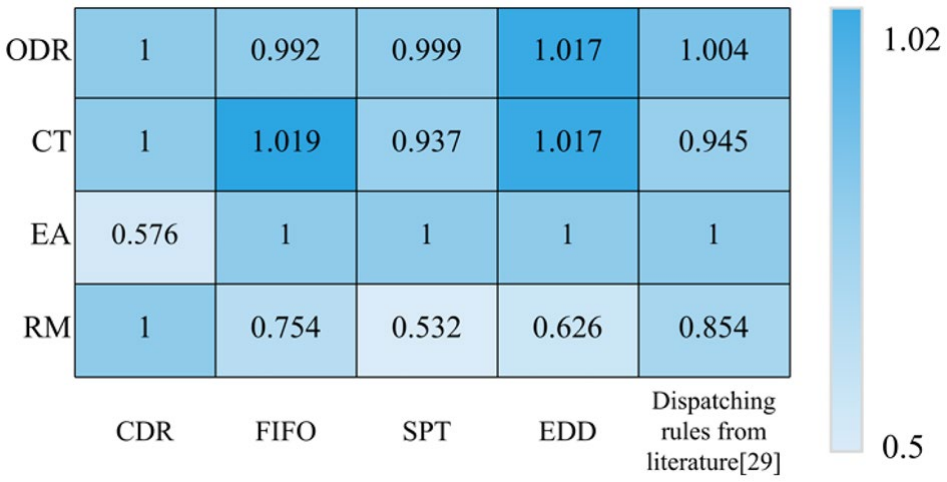
Performances under different dispatch rules.

To further verify the effectiveness of the combined dispatching rules proposed in this chapter, the combined dispatching rules are compared with FIFO, SPT, EDD, and adaptive dispatching rules. The statistics of average CT, ODR, EA, and RM of scheduling rules are respectively made, and the results are shown in Table 3. Since different scheduling parameter combinations will produce different performance index combinations, to avoid the randomness of the experimental results, we set 20 different scheduling parameter combinations and average the experimental results. To compare this dispatching rule with other heuristic dispatching rules more intuitively, the other three dispatching rules are normalized based on the "combined dispatching rules" in Table 3, and the results are shown in Fig. 8.

**Table 3:** Performance indicators under different dispatch rules.

Performances	CDR	FIFO	SPT	EDD	Dispatching rules from literature^29^
CT (h)	42.037	42.853	39.374	42.761	39.738
ODR (%)	82.672	81.974	82.592	84.048	82.974
EA (%)	0.576	1	1	1	1
RM	0.329	0.248	0.175	0.206	0.281

The following conclusions can be obtained by analyzing Table 3 and Fig. 8:

1. The EA index of FIFO, SPT, EDD, and the scheduling rules in^29^ is 1. Because these four scheduling rules do not consider predictive maintenance of equipment, the equipment failure rate is 0, so the EA index is 1.
2. The CT, ODR, and RM of the combined dispatching rule are better than FIFO, because FIFO is first come first processing, which can only reduce the waiting time of the workpiece globally and has poor optimization effect on the overall performance of the system.
3. The ODR and RM of the combined dispatching rule are better than SPT, but the average CT is lower than SPT. This is because SPT preferentially selects the workpieces with short processing time, which can shorten the average CT of the workpieces, but at the same time, it will make the queue length of the workpieces exceed the expectation and may increase the rework rate of the workpieces.
4. The average CT and RM of the combined dispatching rule are better than EDD, but the ODR is lower than EDD. This is because EDD preferentially selects the workpieces with urgent delivery for processing, which can maximize the ODR. However, EDD does not consider the overall status of the production line, so it may not be the most suitable dispatching rule for the current production line status and cannot reflect real-time and dynamic nature.
5. Although the combined scheduling rule is slightly inferior to the scheduling rule described in the literature^29^ in terms of CT and ODR, it can more effectively deal with uncertain events in the production process, so as to more effectively improve production efficiency, maintain machine availability, and avoid deterioration of actual scheduling performance; The scheduling rules described in literature^29^ do not consider equipment failure and preventive maintenance, and cannot reflect the authenticity of the actual scheduling problem.

#### Closed-loop optimization based on IWOA-MLP algorithm

**Figure 9:**
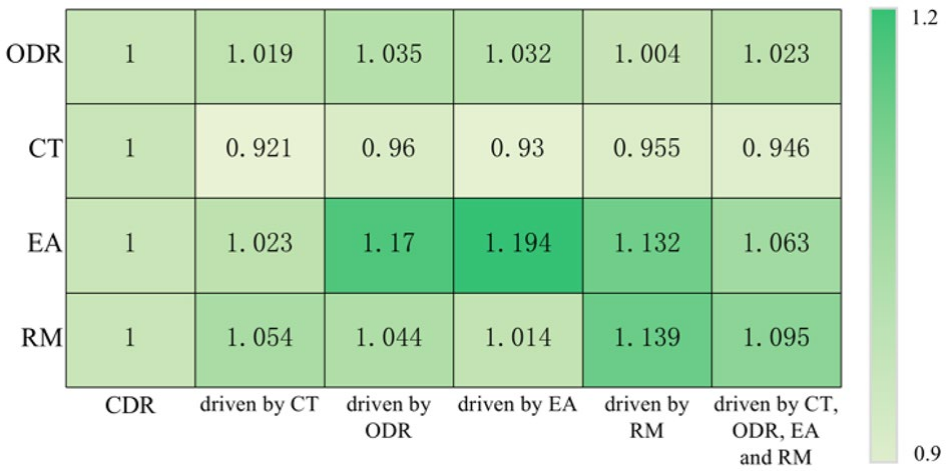
IWOA-MLP based simulation results under different driving sources.

The simulation experiment compares the scheduling system based IWOA-MLP with the compound dispatching rules. As different scheduling parameter combinations will lead to different performance index values, the experimental results are shown in Table 4. Among them, "scheduling rules" refer to the combined scheduling rules that comprehensively consider equipment and workpieces in Section "Composite dispatch rules"; "Closed-loop optimal scheduling system" refers to a robust optimal scheduling system based on IWOA-MLP algorithm, namely "combined scheduling rules + closed-loop optimization based on IWOA-MLP algorithm". To make the simulation results more intuitive, the experimental results are normalized based on the "dispatching rules", and the results are shown in Fig. 9.

**Table 4:** Simulation results under different driving sources.

No	Experiment scenario	Performances			
		CT (h)	ODR (%)	EA (%)	RM
1	CDR	42.037	82.672	0.576	0.367
2	Driven by CT	38.728	84.253	0.589	0.387
3	Driven by ODR	40.362	85.543	0.674	0.383
4	Driven by EA	39.082	85.321	0.688	0.372
5	Driven by RM	40.151	83.046	0.652	0.418
6	Driven by CT, ODR, EA, and RM	39.785	84.589	0.612	0.402

Based on Table 4 and Fig. 9, the following conclusions can be drawn:

1. Compared with the "combined scheduling rules", the "four performance driven closed-loop optimal scheduling system" can improve the CT, ODR, EA and RM performance of the system by 5.357%, 2.319%, 8.491%, and 9.537% respectively. The effectiveness of the "performance driven" and "closed-loop optimization" links are illustrated.
2. Compared with the "combined scheduling rules", the closed-loop optimal scheduling system based on machining cycle drive can improve the CT, ODR, EA, and RM performance of the system by 7.872%, 1.912%, 3.066%, and 5.450%, respectively. The method based on machining cycle drive can greatly improve the machining cycle index of the system, indicating the effectiveness of "performance driven".
3. Similar to conclusion 2), compared with the "combined scheduling rule", the closed loop optimal scheduling system based on ODR drive can greatly improve the ODR performance of the system, while the improvement of other performance is small; The EA driven closed-loop optimal scheduling system can greatly reduce the EA performance of the system; The closed loop optimal scheduling system based on RM drive can greatly improve the RM performance of the system.
4. This part of experiment can show the effectiveness of "performance driven" and "closed-loop optimization" in this method at the same time.

#### Robust optimal scheduling system based on IWOA-MLP algorithm vs. other scheduling systems

**Figure 10:**
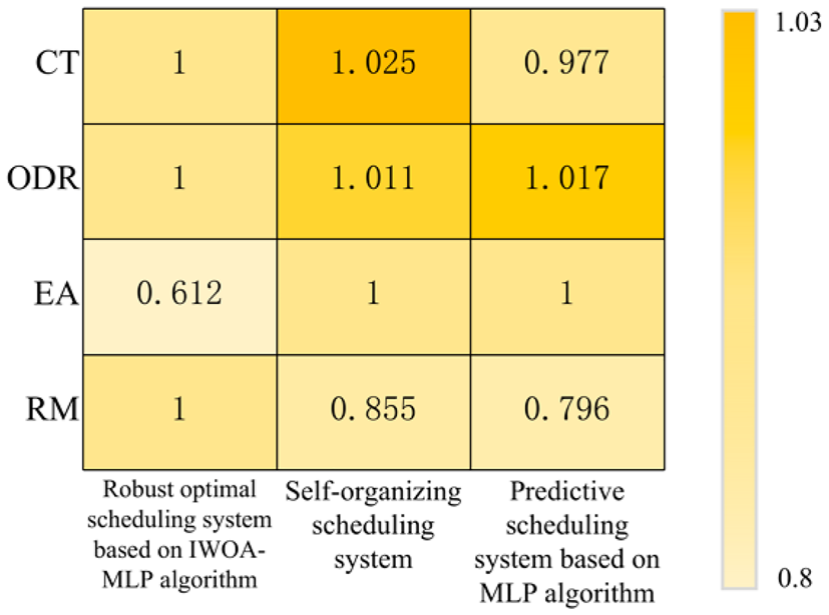
Simulation results of different scheduling systems.

To test the effectiveness of the whole system of "Robust Optimal Scheduling System Based on IWOA-MLP Algorithm" proposed in this paper, it will be compared with the self-organized scheduling system proposed in literature^29^ and the predictive scheduling system based on MLP algorithm proposed in literature^31^. The experimental results are shown in Table 5. To make the results more intuitive, the experimental results of the robust optimal scheduling system based on IWOA-MLP algorithm are used as a benchmark to normalize the experimental results, as shown in Fig. 10.

**Table 5:** Simulation results of different scheduling systems.

Scheduling systems	Performances				
	CT (h)	ODR (%)	EA (%)	RM	Execution time (s)
Robust optimal scheduling system based on IWOA-MLP algorithm	39.785	84.589	0.612	0.442	1189.21
Self-organizing dispatching system	40.784	85.534	1	0.378	682.48
Predictive scheduling system based on MLP algorithm	38.851	86.028	1	0.352	990.89

Based on Table 5 and Fig. 10, the following conclusions can be drawn:

1. This dispatching system integrates equipment failure and equipment preventive maintenance. The other two dispatching systems believe that the equipment is always available. Based on this, the CT and ODR generated by the three scheduling systems are comparable, which is sufficient to illustrate the superiority of the scheduling system.
2. Compared with the self-organized scheduling system and the predictive scheduling system, the robust optimal scheduling system based on IWOA-MLP algorithm can improve the RM of the system by 16.931% and 25.568%, respectively. It shows that the robust optimal scheduling system proposed in this paper has high RM while improving the system performance.
3. The proposed method combines MLP and IWOA and belongs to the deep neural network method, which does require longer execution time. So, compared with the other two scheduling methods, this method requires more time to generate new scheduling schemes. Although there are differences in the execution-time of the three methods, they are all within the real-time requirements of industrial scheduling problems.

## Conclusions

In this research, to improve the performances of the semiconductor manufacturing system, we proposed an IWOA-MLP-based scheduling method with a performance-driven and a feedback-mechanism. We designed a dynamic dispatching rule which integrated equipment maintenance, and process constraints, and correlated the real-time status of the manufacturing system. We used numerous samples obtained from a virtual simulation model of an industrial semiconductor manufacturing system to construct a performance prediction model which can output performance estimation according to 11 processing statuses, which were selected through a feature selection method. In addition, we built a parameter optimization model which can output optimized dispatching parameters according to the 11 processing statuses and four predicted performances. Using the parameter optimization model, we could obtain the most suitable dispatching parameters which can update the dispatching rule to adaptively acclimatize to a new production environment.

The simulation results of this study indicated that the proposed IWOA-MLP-based scheduling method outperformed other several conventional scheduling policies in average cycle time (CT), equipment availability (EA), on-time delivery rate (ODR), and robustness measure (RM). Moreover, it can self-adaptively satisfy the dynamic environment of the manufacturing system and improve the overall performance of the semiconductor manufacturing system.

## Data Availability

As the datasets analyzed during the current study contain secrets of a semiconductor manufacturing company. In most cases, it would not be convenient for the company to disclose such original data, but the corresponding author can provide the data upon request.
